# Immunometabolism in cancer at a glance

**DOI:** 10.1242/dmm.034272

**Published:** 2018-08-02

**Authors:** Katrin Singer, Wan-Chen Cheng, Marina Kreutz, Ping-Chih Ho, Peter J. Siska

**Affiliations:** 1 Department of Internal Medicine III, University Hospital Regensburg, 93053 Regensburg, Germany; 2 Department of Fundamental Oncology, Faculty of Biology and Medicine, University of Lausanne, CH-1066 Epalinges, Vaud, Switzerland; 3 Ludwig Lausanne Branch, CH-1066 Epalinges, Vaud, Switzerland

**Keywords:** Cancer, Metabolism, Metabolites, Nutrients, T cells, Tumor immunology

## Abstract

The scientific knowledge about tumor metabolism has grown at a fascinating rate in recent decades. We now know that tumors are highly active both in their metabolism of available nutrients and in the secretion of metabolic by-products. However, cancer cells can modulate metabolic pathways and thus adapt to specific nutrients. Unlike tumor cells, immune cells are not subject to a ‘micro-evolution’ that would allow them to adapt to progressing tumors that continuously develop new mechanisms of immune escape. Consequently, immune cells are often irreversibly affected and may allow or even support cancer progression. The mechanisms of how tumors change immune cell function are not sufficiently explored. It is, however, clear that commonly shared features of tumor metabolism, such as local nutrient depletion or production of metabolic ‘waste’ can broadly affect immune cells and contribute to immune evasion. Moreover, immune cells utilize different metabolic programs based on their subtype and function, and these immunometabolic pathways can be modified in the tumor microenvironment. In this review and accompanying poster, we identify and describe the common mechanisms by which tumors metabolically affect the tumor-infiltrating cells of native and adaptive immunity, and discuss how these mechanisms may lead to novel therapeutic opportunities.

## Abstract (precis)

**Summary:** This ‘At a Glance’ review and accompanying poster address how tumors can negatively affect immune cells through depletion of critical nutrients or through production of toxic metabolic products.

## Introduction

It has long been known that cancer cells hijack cellular programs that regulate survival, growth and proliferation, leading to tumor formation and progression. The best-known causes of malignant transformation are the genetic and epigenetic changes that induce stem-cell-like properties, such as unlimited cell division and blocked differentiation. Traditionally, the proposed role of the cellular metabolism of cancer cells was to primarily support and sustain malignant growth. However, it is clear today that cellular metabolism actively regulates the malignant phenotype. For example, loss of the p53 tumor suppressor may contribute to malignant transformation independently of its well-described functions in cell cycle regulation, DNA repair and senescence (see Box 1 for a glossary of terms). Instead, through the induction of glycolysis and anabolic pathways (Box 1), p53 dysfunction leads to an early-onset metabolic malignant transformation (Li et al., 2012). Another example of a key role of a mutation-driven metabolic reprogramming leading to malignant transformation are oncometabolites. For example, a consequence of loss-of-function mutations in succinate dehydrogenase (SDH; Box 1) is that cancer cells can massively accumulate succinate, an intermediate metabolite in the tricarboxylic acid (TCA) cycle. Intriguingly, succinate, now in the role of an oncometabolite, can induce epigenetic alterations through the inhibition of α-ketoglutarate-dependent dioxygenases (Xiao et al., 2012), ultimately leading to a malignant phenotype (Wong et al., 2017). Some features of metabolic reprogramming in cancer have, however, been puzzling, such as the Warburg effect, in which tumor cells increase glucose consumption and lactate excretion (Warburg et al., 1927). This phenotype is energetically inefficient compared to mitochondrial respiration, and could theoretically limit tumor growth in glucose-depleted tissues. However, anaerobic glycolysis can be beneficial if the malignant cell requires a high metabolic flux to synthesize building blocks such as nucleotides. Moreover, this phenotype can induce a unique metabolic milieu with low glucose and high lactate (Chang et al., 2015; Jang et al., 2013; Siska and Rathmell, 2015). Intriguingly, evidence from murine *in vitro* and *in vivo* models suggests that glucose deprivation and lactate accumulation in the tumor microenvironment can have detrimental effects on the immune cells that were poised to infiltrate and destroy tumors (Cham et al., 2008; Chang et al., 2015).

**Figure:**
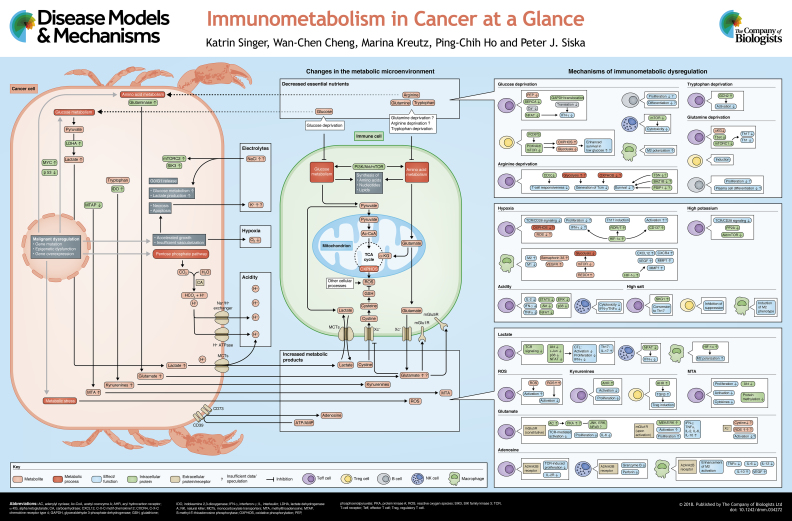

**Box 1. Glossary**

**^13^C-labeling:** method to interrogate intracellular metabolic pathways. Detection of labeled metabolites is performed using nuclear magnetic resonance spectroscopy.

**Anabolic pathways:** synthesis of macro-molecules out of smaller biochemical components.

**CD4+ T cells:** T cells expressing CD4. Often referred to as ‘helper’ T cells; can differentiate to inflammatory (‘effector’) and anti-inflammatory (‘regulatory’) subtypes.

**CD4+ Treg cells:** CD4+ T cells with regulatory properties. Usually described by high CD25 and FOXP3 expression. Critical for maintenance of self-tolerance.

**CD8+ T cells:** T cells expressing CD8. Often referred to as ‘cytotoxic’ T cells; capable of direct engagement with infected cells or tumor cells.

**Chimeric antigen receptor (CAR)-transduced T cells:** engineered effector T cells, recognizing specific antigens expressed by tumor cells, such as CD22 in B-cell leukemia.

**Costimulatory receptors:** in addition to T-cell receptor (TCR) stimulation, ligation of costimulatory receptors such as CD28, CD137 and ICOS increases or modulates T-cell activation.

**Germinal center:** area in lymphoid follicles where B cells become activated, proliferate intensively after antigen contact, switch immunoglobulin class and increase affinity for the antigen.

**Granzyme-B and perforin:** cytolytic molecules stored in the granules of cytotoxic T cells and natural killer (NK) cells.

**Immune checkpoint inhibitors:** monoclonal antibodies that block immune inhibitory pathways such as CTLA-4, PD-1 and PD-L1, and induce immune-cell activation.

**Interferon-ɣ (IFN-ɣ):** inflammatory cytokine, mainly produced by T cells and NK cells, with anti-tumoral, anti-viral and immunostimulatory properties.

**L-kynurenine:** product of L-tryptophan degradation through tryptophan dioxygenase and indoleamine 2,3-dioxygenase.

**Lymphoid/lymphatic organs:** spleen, bone marrow, thymus, appendix, lymph nodes, lymph vessels and tonsils. Critical for formation, maturation, differentiation and activation of immune cells.

**Myeloid-derived suppressor cells (MDSCs):** heterogeneous population of immature myeloid cells consisting of precursors for granulocytes, macrophages or dendritic cells. Associated with resolution of inflammation and tumor progression.

**Pentose phosphate pathway (PPP):** series of metabolic steps leading to degradation of glucose to pentoses via the formation of NADPH and carbon dioxide.

**Plasma cells:** differentiated B cells capable of antibody production and secretion.

**Programmed death 1 (PD-1) receptor:** surface protein on activated T cells repressing an immune response. Activated through PD-1 ligands (PD-L1, PD-L2), which are expressed in various tissues, including tumors.

**Retinoic acid receptor-related orphan receptor gamma (RORɣt):** ligand-dependent transcription factor expressed only in cells of the lymphoid compartment, typically in CD4+ T cells secreting IL-17 (Th17 cells).

**Senescence:** age-related alterations in all stages of immune-cell development.

**Succinate dehydrogenase (SDH):** also known as respiratory complex II; catalyzes the oxidation of succinate to fumarate with the reduction of ubiquinone to ubiquinol.

**Toll-like receptor (TLR) ligands:** ligands to the pattern recognition receptors and activator of innate immune cells; e.g. microbial cell wall components (e.g. lipopolysaccharide) and viral molecules.

**Tumor-draining lymph nodes:** closest lymph nodes to the tumor. Typically a primary site of tumor dissemination.

Cancers are highly diverse and, in addition to the genetic and functional heterogeneity of malignant cells, a broad spectrum of immune populations can be found in human tumor tissue. Among adaptive immune cells, the tumor-infiltrating T cells are the best documented. T cells are highly heterogeneous, and various phenotypic sub-populations [CD4+ and CD8+ T cells (Box 1)] and functional (effector, memory) and differentiation [CD4+ Th1, CD4+ Treg (Box 1)] states have been identified. T cells can affect tumor growth either through direct engagement or through stimulation of other cells in the tumor microenvironment. This feature has been exploited in clinical approaches that aim to increase their anti-tumor potential, such as through blockade of the T-cell-inhibitory PD-1 receptor (Box 1), or through employment of *ex vivo* engineered chimeric antigen receptor (CAR)-transduced T cells (Box 1). The tumor infiltration with B cells is less well documented, but both their pro- and anti-tumorigenic functions (Tsou et al., 2016) are intriguing and require extensive elucidation in future studies.

The interaction of adaptive immune cells with cells of innate immunity is critical for an effective and well-regulated response, and innate immune cells are often found in tumors. Indeed, the first immune cells to be described in human tumors were macrophages (Lewis and Pollard, 2006). Outside of the context of cancer, these innate immune cells are responsible for fast clearance of pathogen-infected cells during infections. Upon stimulation with interferon-γ (IFN-γ) and toll-like receptor (TLR) ligands (Box 1), macrophages polarize to a pro-inflammatory (M1) phenotype, with an anti-tumor potential (Yuan et al., 2015). Additionally, macrophages can also polarize toward an anti-inflammatory phenotype with pro-tumoral characteristics through alternative activation (M2) when stimulated with IL-4 and IL-10. M1 and M2 macrophages participate in inflammatory responses and modulate tissue homeostasis and repair through their distinct functional specialties (Martinez and Gordon, 2014). Hence, macrophages must be highly plastic to adapt their functions in response to infection and tissue damage. Emerging evidence reveals that macrophages engage distinct metabolic demands during M1 and M2 activation. For example, M1 macrophages enhance their anabolic metabolism, including anaerobic glycolysis, pentose phosphate pathway (Box 1) activation and fatty acid synthesis. In contrast, M2 macrophages prefer catabolic metabolism and heavily utilize oxidative phosphorylation (OXPHOS) to support their metabolic demands (Ho and Liu, 2016). These changes provide metabolic checkpoints to fine-tune macrophage behavior and contribute to their altered functions in diseases, especially in the tumor microenvironment. Also part of the innate immunity, natural killer (NK) cells are critical for direct engagement and killing of cells identified as non-self. Accordingly, they have the potential to destroy cancer cells (Marcus et al., 2014). Compared to T cells and macrophages, NK cell metabolism is less well documented. Accordingly, if and how the tumor microenvironment affects NK cell metabolism is mostly unknown. We hypothesize, however, that several of the key mechanisms of metabolic immune cell suppression in tumors are shared between the various immune cell types that infiltrate tumors.

## Tumors modulate local concentrations of nutrients that are critical for immune cell function

### Glucose metabolism

To perform effector functions, including killing cancer cells and excreting cytokines, effector immune cells, such as activated cytotoxic T cells, undergo extensive metabolic reprogramming. Aerobic glycolysis, a process where glucose is metabolized to pyruvate and ultimately to lactate in a series of enzymatic steps that yield ATP and substrates for other metabolic pathways, was first described in malignant cells. Interestingly, non-malignant proliferating cells perform aerobic glycolysis, which is also considered necessary for optimal T-cell function (Cham et al., 2008). However, T cells are metabolically flexible and aerobic glycolysis may not be critical for their activation and survival (Chang et al., 2013; Renner et al., 2015). In contrast, glycolysis is essential for T-cell proliferation (Renner et al., 2015). Under normoglycemic conditions, T cells upregulate the key glucose transporter Glut1, followed by increased glucose uptake and glycolysis upon antigenic stimulation (Frauwirth et al., 2002; Macintyre et al., 2014). In parallel, pyruvate, one of the terminal products of glycolysis, is mainly reduced to lactate, rather than being oxidized in mitochondrial respiration (see poster). However, T cells may not be able to sustain a permanent state of anaerobic glycolysis that is necessary for their effector function. During acute infections, the population of effector T (Teff) cells performing aerobic glycolysis contracts and memory T cells, which are less dependent on glycolysis and rather engage in mitochondrial respiration, arise (Buck et al., 2015; MacIver et al., 2013).

In contrast, cancer cells are able to maintain and eventually increase high glucose uptake and glycolysis, leading to a decrease of intratumoral glucose levels (Busk et al., 2011; Voelxen et al., 2017; Battista et al., 2016). In turn, glucose deprivation can directly impede production of IFN-γ, a key T-cell effector molecule in tumor-infiltrating CD8+ T cells (Chang et al., 2015). It has been proposed that these effects are mediated through glyceraldehyde-3-phosphate dehydrogenase (GAPDH), a key glycolytic enzyme that can also affect post-transcriptional modification of mRNA. When T cells are able to perform high rates of glycolysis, GAPDH is committed to its metabolic role. Under low glycolytic flux, however, GAPDH prevents translation of IFN-γ (Chang et al., 2013). It has also been documented that glucose deprivation suppresses T cell receptor (TCR)-dependent activation of Ca^2+^ and nuclear factor of activated T cells (NFAT) signaling, leading to T-cell hypo-responsiveness. This effect is mediated by the absence of the glycolytic product phosphoenolpyruvate, which sustains Ca^2+^ and NFAT by blocking sarco/endoplasmic reticulum Ca^2+^-ATPase (Ho et al., 2015). In line with this, our group observed a negative correlation between accelerated tumoral glucose metabolism and T-cell infiltration in renal cell carcinoma (Singer et al., 2011), with similar observations made in oral squamous cell carcinoma (Ottensmeier et al., 2016). Interestingly, emerging clinical data point towards tumor glucose metabolism as a mechanism of resistance to T-cell-mediated tumor rejection. As recently shown by Cascone et al., overexpression of glycolysis-related genes in cancer cells impairs the anti-tumor activity of T cells and, inversely, the inhibition of cancer glycolysis enhanced T-cell-mediated tumor rejection (Cascone et al., 2018).

CD4+ regulatory T (Treg) cells can suppress inflammation and are often associated with tumor progression. Tumor-infiltrating Treg cells may inhibit local anti-tumor immunity. Interestingly, as described in murine systems, Treg cells express low levels of Glut1, do not rely on glucose uptake and glycolysis, and, similarly to CD8+ memory T cells, perform OXPHOS and lipid oxidation (Michalek et al., 2011). Forkhead box protein P3 (FOXP3), the lineage-defining transcription factor of murine Treg cells, was proposed to be a key regulator of this phenotype (Gerriets et al., 2016). Mechanistically, FOXP3 induces the expression of genes involved in lipid and peptide hormone metabolism. Additionally, it downregulates genes involved in glucose uptake and glycolysis. Importantly, forced expression of FOXP3 inhibits the PI3K-Akt-mTORC1 signaling pathway, which is involved in the induction of the glycolytic machinery (Gerriets et al., 2016). Paradoxically, the abundance of glucose may be important for Treg induction, as glycolysis in conventional CD4+ T cells is essential for the initiation of the regulatory phenotype in Treg cells through the translocation of the glycolytic enzyme enolase-1 to the nucleus, where it binds to *FOXP3* regulatory regions, such as its promoter and its CNS2 (conserved noncoding sequence 2) (De Rosa et al., 2015). These studies suggest that glucose is necessary for the emergence of Treg cells, e.g. in a lymphatic organ (Box 1), and, upon a subsequent tumor infiltration, glucose may favor Treg survival and function. However, future studies are required to address this hypothesis in cancer patients.

Similar to T cells, B cells are highly metabolically active. During B-cell development, each stage has a different dependency on glucose metabolism and pre-B-cells are less glucose-dependent than immature B cells (Kojima et al., 2010). After stimulation, naïve B cells proliferate and increase glucose uptake and lactate production, similarly to what occurs upon T-cell activation (Garcia-Manteiga et al., 2011). In line, extensive B-cell metabolic reprogramming is required for antibody production (Caro-Maldonado et al., 2014). Recently, Jellusova et al. described the requirement of high glycolytic activity of germinal center (Box 1) B cells to support their growth and proliferation in a hypoxic microenvironment. In addition to glycolysis, an increase of mitochondrial content has been observed in germinal center B cells (Jellusova et al., 2017). We speculate that glucose deprivation in combination with hypoxia in the tumor microenvironment might favor certain B-cell phenotypes, creating an immunosuppressive milieu.

Resembling the features of Teff cell metabolism, NK cells increase aerobic glycolysis upon activation (Gardiner and Finlay, 2017). With high IL-15 stimulation, NK cells elevate the activity of mammalian target of rapamycin (mTOR) to boost bioenergetic metabolism, increase glucose uptake, and upregulate the expression of transferrin receptor CD71 and amino acid transporter CD98 (Marcais et al., 2014). Accordingly, impairment of glucose metabolism and disruption of mTOR signaling leads to a diminished cytotoxic activity in NK cells (Donnelly et al., 2014). Assmann et al. recently showed that sterol regulatory element-binding protein (Srebp) transcription factors play an essential role in the cytokine-induced metabolic reprogramming of NK cells. Srebp was required for the increase in both glycolysis and OXPHOS. Moreover, Srebp inhibition prevented this phenotype and decreased NK cell cytotoxicity (Assmann et al., 2017). It remains unclear, however, whether metabolic alterations found in tumors may affect the metabolic activity and the Srebp-mediated NK cell function.

### Amino acid metabolism

In addition to glucose, glutamine has been described as a crucial nutrient for the effector function of T cells. Glutamine is the most abundant amino acid in circulation and its uptake is critical for various T-cell metabolic processes, including the TCA cycle, nucleotide synthesis and detoxification of reactive oxygen species (ROS) (Johnson et al., 2016). As shown by Nakaya et al., T-cell glutamine uptake depends on the neutral amino acid transporter type 2 (ASCT2). ASCT2 deficiency blocks the induction of T helper 1 (Th1) and Th17 cells (Nakaya et al., 2014). In line with this, it has been reported that glutamine deprivation supports differentiation into Tregs, despite *in vitro* conditions favoring a Th1 differentiation. Moreover, addition of α-ketoglutarate reversed this effect and rescued Th1 differentiation under glutamine deprivation through the induction of Tbet, a Teff cell transcription factor, which correlated with increased mTORC1 signaling (Klysz et al., 2015). As reported by Lee et al., 6-diazo-5-oxo-L-norleucine, a naturally occurring glutamine antagonist, inhibited glutamine metabolism in activated T cells and was able to inhibit immune-mediated rejection of allografts in fully mismatched skin and heart allograft transplantation models (Lee et al., 2015). Similarly, glutamine is essential for B-cell proliferation and differentiation into plasma cells (Box 1) (Crawford and Cohen, 1985)*.* Not much is known about glutamine concentrations in tumors, but many cancer types harbor mutated Myc, leading to high glutamine uptake (Gao et al., 2009). Myc transcriptionally induces mitochondrial glutaminolysis and leads to glutamine addiction of cancer cells (Wise et al., 2008). Thus, glutamine may be limited in the tumor environment and glutamine deprivation can play a role in tumor-induced immunosuppression.

Upon activation, T cells heavily consume arginine and tryptophan (Sinclair et al., 2013). L-arginine enhances the generation of central memory-like T cells by inducing a metabolic switch from glycolysis to OXPHOS, with enhanced anti-tumor activity in an OVA-antigen-expressing B16 melanoma mouse model (Geiger et al., 2016). The authors suggest that L-arginine has a direct effect on specific T-cell nuclear proteins (BAZ1B, PSIP1 and TSN) by changing their structure, leading to increased pro-survival signaling and enhanced anti-tumor function in T cells (Geiger et al., 2016).

However, cancer cells often overexpress the amino-acid-catabolic enzyme indolamine-2,3-dioxygenase (IDO), which can lead to extracellular depletion of tryptophan (see poster, cancer cell). Constitutive expression of IDO depends on cyclooxygenase-2 and prostaglandin E2 via PKC and PI3K signaling (Hennequart et al., 2017). Similar to glucose, deprivation of tryptophan can impair T-cell function. Specifically, tryptophan depletion activates general control nonderepressible 2 (GCN2), a stress-response kinase that is activated by elevations in uncharged transfer RNA (tRNA), leading to inhibition of T-cell function (Munn et al., 2005), impaired Th17 differentiation and promotion of Treg development (Sundrud et al., 2009). Similar to IDO, tryptophan 2,3-dioxygenase (TDO) is expressed by cancer cells in various human tumors (Pilotte et al., 2012) and its activity, presumably through tryptophan depletion or kynurenine (Box 1) production, induces immune dysfunction (Schmidt et al., 2009). As shown by Pilotte et al., TDO inhibition through a novel synthetic inhibitor was able to restore the ability of mice to reject TDO-expressing tumors in a preclinical mastocytoma model (Pilotte et al., 2012).

Additionally, degradation of arginine by tumors or myeloid-derived suppressor cells (Box 1) through arginase-1 upregulation leads to reduced expression of the CD3ζ chain, cell cycle arrest and an impaired antigen-specific T-cell response (Rodriguez et al., 2007). In line with these observations, tumor cells overexpressing IDO were not rejected by tumor-specific T cells in a P815 mastocytoma murine model (Uyttenhove et al., 2003), further confirming the immunoregulatory role of amino acid catabolism in cancer.

### Oxygen

Tumors are often hypoxic, as malignant growth can exceed the capacity of healthy progenitor cells to form new blood vessels. In turn, hypoxia can function as a metabolic adjunct to further promote a malignant phenotype. Indeed, hypoxia can boost glucose uptake and glycolysis through induction of various glycolytic genes (Kim et al., 2006; Semenza et al., 1996), and elevated glycolysis is associated with sustained malignant growth (Jang et al., 2013). The effects of hypoxia on immune cell activation are not sufficiently explored. On the one hand, hypoxic conditions lead to less efficient TCR- and CD28-mediated T-cell activation (Neumann et al., 2005). Moreover, hypoxia-inducible factor (HIF)-1α-deficient CD4+ and CD8+ T cells from Lck-Cre/HIF-1-floxed mice show an increased ability to proliferate and to produce IFN-γ (Lukashev et al., 2006). On the other hand, it has been shown that HIF-1α does not affect T-cell proliferation, but favors the differentiation of Th17 cells via direct transcriptional induction of the RAR-related orphan receptor gamma (RORγt; Box 1) (Dang et al., 2011). Interestingly, hypoxia-induced HIF-1α is able to increase the expression of costimulatory receptors (Box 1), such as CD137, on tumor-infiltrating T cells (Palazon et al., 2012). Hypoxia may therefore be selectively required for effective immunotherapies that aim to stimulate the anti-tumor activity of T cells.

Oxygen is necessary for OXPHOS and the generation of ROS. Both processes are part of mitochondrial respiration and ROS are necessary for proper T-cell effector function and antigen-specific proliferation (Sena et al., 2013). The effects of ROS on CD8+ T-cell function are mediated by lymphocyte expansion molecule (LEM), which regulates the expression of OXPHOS proteins (such as NADH ubiquinone oxidoreductase chain 1) and, accordingly, ROS production. LEM is necessary for cytotoxic T-cell expansion and memory T-cell development (Okoye et al., 2015). Therefore, basal ROS levels are required for proper T-cell signaling. Under hypoxia, ROS levels may be insufficient. Conversely, high ROS levels can be toxic and the ROS generated in the tumor microenvironment can impair immune cells by downregulation of the CD3ζ chain or impairment of Ca^2+^ mobilization (Ando et al., 2008; Kono et al., 1996). ROS induce oxidation of lipids such as 4-hydroxynonenal (4-HNE), inducing apoptosis as well as defects in NFκB signaling in T cells (Liu et al., 2001). Additionally, 4-HNE activates X-box binding protein 1 in tumor-associated dendritic cells (DCs), leading to an accumulation of lipid bodies driving ovarian cancer progression via the suppression of anti-tumoral immune responses (Cubillos-Ruiz et al., 2015). In line with this, high intracellular levels of ROS derived from mitochondria may impair anti-tumoral T-cell function (Siska et al., 2017).

Macrophages are sensitive to changes in oxygen availability and it has been reported that the anti-inflammatory M2-like tumor-associated macrophages (TAMs) accumulate in hypoxic tumor regions, whereas the pro-inflammatory M1-like TAMs reside in normoxic regions. Mechanistically, intratumoral hypoxia-induced semaphorin 3A (Box 1) attracts TAMs to hypoxic regions by triggering vascular endothelial growth factor (VEGF) receptor 1 phosphorylation (Casazza et al., 2013). In addition, hypoxic TAMs upregulate the expression of REDD1 (regulated in development and DNA damage responses 1), which inhibits mTOR. This leads to decreased glycolysis in TAMs and correlates with further hypoxia by abnormal blood vessel formation and promotion of metastases (Wenes et al., 2016). Moreover, hypoxia stabilizes HIF-1α in TAMs, leading to high production of chemokines and chemokine receptors such as C-X-C motif chemokine ligand 12 (CXCL12) and receptor 4 (CXCR4) (Schioppa et al., 2003), as well as VEGF (Forsythe et al., 1996). Hypoxic TAMs also secrete proteolytic enzymes, such as matrix metalloproteinases (MMP)-1 (Murdoch and Lewis, 2005) and -7 (Burke et al., 2003). Production of metalloproteinases by TAMs is likely to affect the interaction of both endothelial and tumor cells with the extracellular matrix, contributing to cell proliferation and tumor dissemination.

## Waste products of tumor metabolism affect immunity

### Glucose metabolism and acidity

Although the concentration of essential nutrients may be lower in the tumor microenvironment compared to normal tissues, several waste products of tumor cell metabolism accumulate and can affect immune cell function. The most prominent metabolite in the tumor microenvironment is lactate. After the reduction of pyruvate to lactate, the monocarboxylate transporters (MCT)-1 and -4 co-transport lactate and protons out of the cell, leading to an accumulation of lactate and to a decreased pH in the extracellular space (see poster, cancer cell). Accordingly, intratumoral lactate can reach levels of up to 40 mM (Brand et al., 2016), and high intratumoral lactate concentrations correlate with a more aggressive tumor biology and decreased patient survival in some cancers, such as in head-and-neck tumors and melanoma (Brizel et al., 2001; Brand et al., 2016).

To date, several studies demonstrated strong effects of lactate and lactic acid on immune cell populations *in vitro* and *in vivo*. For example, lactate/lactic acid promoted IL-17 production by CD4+ Th17 cells (Haas et al., 2015), while inhibiting proliferation and activation of cytotoxic CD8+ T cells (Fischer et al., 2007). This effect is induced by the prevention of TCR-triggered phosphorylation of JNK, c-Jun and p38, as well as the expression of NFAT (Mendler et al., 2012; Brand et al., 2016). In line with this, our group showed that lactate dehydrogenase A (LDHA)-mediated production of lactate in tumor cells and subsequent acidification constrains IFN-γ production in tumor-infiltrating T cells, resulting in a loss of immune surveillance and promoting tumor growth in a mouse melanoma model (Brand et al., 2016). Importantly, ^13^C-labeled (Box 1)-lactate uptake experiments demonstrated that protons are required for the effects of lactate/lactic acid on immune cells, since the addition of protons could increase lactate uptake into the immune cells (Brand et al., 2016; Fischer et al., 2007). Innate immune cells are also sensitive to lactate. High amounts of lactate in the tumor microenvironment stimulate TAM polarization into the M2-like phenotype by stabilizing HIF-1α (Colegio et al., 2014). In addition, high levels of lactic acid in tumors downregulate NK cell activation, resulting in diminished IFN-γ production and tumor immune escape (Brand et al., 2016).

Although the negative impact of lactate on immune cells is often in concert with a decreased pH in the tumor microenvironment, acidity has distinct effects on a variety of immune populations. Tumors can be highly acidic, and it has been extensively reported that low pH supports cancer growth and spreading (Kato et al., 2013). Proton secretion from tumor cells to induce extracellular acidity can be carried out by several transporters, such as the Na^+^/H^+^ exchanger, the above-mentioned lactate/H^+^ co-transporting MCTs and the H^+^ ATPase. In addition, intratumoral hypoxia can induce carbonic anhydrase, which can form protons by catalyzing the hydration of CO_2_ (Ivanov et al., 2001). In line with this, increased CO_2_ production through the pentose phosphate pathway in cancer cells has been linked to an acidification of the tumor microenvironment (Kato et al., 2013). As broadly reviewed by Huber et al. (2017), low pH negatively impacts the effector functions of both innate and adaptive immune cells. This was first described by Fischer et al., who demonstrated that low extracellular pH (pHe: 5.8) leads to decreased cytokine production and to a loss of cytotoxic effector functions without affecting cell viability (Fischer et al., 2000a,b; Muller et al., 2000). The effect of low pH on cytokine production by T cells correlated with impaired signaling pathways involving STAT5, ERK, AKT, p38 and NFAT (Huber et al., 2017). Importantly, buffering of low pH with bicarbonate therapy increased T-cell infiltration and impaired tumor growth. Furthermore, a combination of bicarbonate with anti-CTLA4 and anti-PD-1 (Box 1) treatments improved antitumor responses in B16 melanoma and Panc02 pancreatic cancer mouse models and increased the survival of mice in a pmel-B16 model of adoptive T-cell therapy (Pilon-Thomas et al., 2016). Despite these results, a specific acidity-sensing machinery in T cells and other immune cells still needs to be identified.

### Amino acid metabolism

In addition to tryptophan depletion, high activity of IDO leads to an accumulation of tryptophan catabolism byproducts, most prominently kynurenines. Similarly to lactate, kynurenines can suppress the proliferation and the effector function of CD8+ T cells through the aryl hydrocarbon receptor (AHR) (Opitz et al., 2011; Fallarino et al., 2006). Interestingly, the interaction of kynurenines with AHR favors Treg induction in a TGFβ-dependent manner (Mezrich et al., 2010). The combination of tryptophan starvation and accumulation of tryptophan catabolites downregulates TCRζ and induces a regulatory phenotype in naïve T cells.

Similarly, overexpression of glutaminase, observed in many cancers, might not only decrease glutamine levels, but could lead to high intratumoral glutamate levels. Indeed, Briggs et al. showed that breast cancer cells secrete glutamate, leading to paracrine induction of HIF-1α via inhibition of the glutamate/cystine antiporter Xc^−^ and HIF-PH2 inactivation (Briggs et al., 2016). In addition, macrophages and DCs, which are often found in tumors and tumor-draining lymph nodes (Box 1), can release glutamate in concentrations up to 30 µM (Pacheco et al., 2006). Even though the minimal effective concentration necessary to affect immune cells is unknown, T cells constitutively express the glutamate transporter mGlu5R. mGlu5R stimulates adenylate cyclase and prevents TCR-mediated T-cell activation and IL-6 production (Pacheco et al., 2004, 2006), possibly through the activation of protein kinase A, which can inhibit several pathways, including ERK, JNK and NFκB signaling (Pacheco et al., 2007). However, upon stimulation, T cells express mGlu1R, which signals through the MEK-ERK1/2 pathway, and this counteracts T-cell inhibition through mGlu5R (Pacheco et al., 2004). mGlu1R stimulation by glutamate enhances the secretion of several cytokines, including IL-2 and IFN-γ, and increases proliferation (Pacheco et al., 2006). A high concentration of extracellular glutamate can also affect other transporters, such as Xc^−^. Our group and others have shown that T cells express Xc^−^ and that T-cell stimulation leads to an increased uptake of cystine. Cystine uptake, followed by its intracellular degradation to cysteine and subsequent glutathione synthesis, is critical for the ROS detoxification machinery, and inhibition of cystine uptake impairs T-cell activation (Siska et al., 2016). High levels of extracellular glutamate might therefore impair the export of glutamate and the import of cystine, possibly leading to ROS dysregulation and T-cell dysfunction (see poster, Increased metabolic products). Glutamate receptors have also been found on other immune cells, including B cells and DCs (Pacheco et al., 2007), and future studies of the intratumoral glutamine/glutamate homeostasis may therefore uncover intriguing new mechanisms of tumor-induced immune dysregulation.

### Nucleotide metabolism

In addition to the direct effects of hypoxia on intratumoral immune cells, hypoxia induces increased adenine nucleotide breakdown through the 5′-nucleotidase pathway, leading to adenosine accumulation in tumors (Blay et al., 1997). Specifically, ATP is rapidly degraded to adenosine by the ectonucleotidases CD39 (Eltzschig et al., 2009) and CD73 (Synnestvedt et al., 2002) expressed on tumor cells, which convert ATP to AMP and AMP to adenosine, respectively. Adenosine can in turn inhibit the activation and cytotoxic capacity of T and NK cells (Huang et al., 1997; Hausler et al., 2011). The accumulated extracellular adenosine then binds to A2AR and A2BR adenosine receptors expressed by T cells and NK cells, inducing intracellular cAMP accumulation and signaling (Ohta and Sitkovsky, 2001). Even at low levels, adenosine strongly inhibits both TCR-induced proliferation of T cells and IL-2 receptor expression (Huang et al., 1997). Intriguingly, A2AR and A2BR blockade can enhance NK cell function by increasing granzyme-B (Box 1) expression, and promotes the anti-metastatic effects of NK cells by secreting perforin (Box 1) (Beavis et al., 2013; Mittal et al., 2014). These data suggest that the adenosine pathway also contributes to NK cell dysfunction in the tumor microenvironment.

Similar to its effects on T and NK cells, adenosine enhances activation of immunoregulatory M2 macrophages via A2AR and A2BR, inhibits TNFα, IL-6 and IL-12 release, and augments IL-10 as well as VEGF production (Csoka et al., 2012). Lastly, many tumors show a deficiency in S-methyl-5′-thioadenosine phosphorylase (MTAP). MTAP is responsible for the breakdown of S-methyl-5′-thioadenosine (MTA) and, because of MTAP deficiency, MTA levels can increase in the tumor microenvironment. MTA inhibits antigen-specific T-cell proliferation, activation and cytokine production by interfering with asymmetric protein methylation events upon T-cell stimulation and through decreased Akt phosphorylation (Henrich et al., 2016). Subsequently, MTA-treated T cells do not upregulate the expression of molecules such as CD25 and CD69, and maintain a naïve phenotype. Functionally, highly activated cytotoxic T cells are still not able to lyse target cells and to produce IFN-γ, resulting in a loss of their anti-tumoral capacity. Thus, MTA might hamper T-cell signaling, rendering anti-tumoral T cells unresponsive.

Importantly, the relevance of adenosine-mediated immune dysfunction has been tested in several studies. For example, dual targeting of A2AR and CD73 showed a significant combination benefit in controlling tumor growth and lung metastases in mice (Young et al., 2016). These promising results led to the initiation of several clinical trials with small-molecule inhibitors targeting A2AR (Vijayan et al., 2017). However, more data need to be collected to prove the feasibility of such approaches in cancer patients.

## Salts and other factors as overlooked players in the tumor microenvironment

In addition to glucose, amino acids and other well-researched metabolic substrates, nutrients such as fatty acids and complex lipids might play an immunometabolic role in the tumor microenvironment (Al-Khami et al., 2017; Michalek et al., 2011). Interestingly, vitamins and trace elements are also used by immune cells (Mora et al., 2008; Wintergerst et al., 2007). Therefore, disturbances in the pathways that involve various nutrients and soluble factors may play a role in tumor-induced immune-cell modulation. Not much is known about these pathways, even in a non-cancer setting, and future research to address this area might provide new interesting targets for fine tuning of immune therapy. Interestingly, high salt [sodium chloride (NaCl)] concentrations have recently been linked to enhanced growth, increased glucose consumption and lactate production in cancer cells (Amara et al., 2016; Amara and Tiriveedhi, 2017). In addition, epidemiological studies demonstrated that high salt is correlated with increased incidence of gastric cancer (Tsugane et al., 2004). Studying breast cancer cells, Amara et al. observed a proliferative effect of salt on cancer cells that appeared to be mediated through a G0/G1 phase release following phosphorylation of salt-inducible kinase 3 (SIK3) through mTORC2 (Amara et al., 2017). However, other studies reported that high salt concentrations are anti-proliferative for cancer cells (Arimochi and Morita, 2005). Although there is very limited information about electrolyte concentrations in the tumor microenvironment, given the high proliferative rate, extensive metabolic reprogramming, overexpression of several ion transporters and dysregulated vascularization of tumors, it would not be surprising to find that salt homeostasis is disturbed in malignant tissues.

Interestingly, increased salt concentrations may also affect immune cells. In this context, high NaCl conditions triggered a switch to an inflammatory Th17 phenotype by inducible salt-sensing kinase SGK1 (Wu et al., 2013). In contrast, NaCl inhibited the suppressive function of Treg cells (Hernandez et al., 2015). In a recent cancer vaccine study, an increased salt concentration in the vaccine formulation dramatically improved vaccination-induced tumor rejection through CD8+ T cells in a mouse E.G7-OVA lymphoma model (Luo et al., 2017). In contrast to the pro-inflammatory effects of salt that might support anti-tumor immunity, it has been reported that salt can induce the pro-tumor M2 phenotype of macrophages (Amara and Tiriveedhi, 2017). Potassium (K^+^) is highly abundant intracellularly and is released upon cell death. In a recent paper, high extracellular K^+^ concentrations were detected in necrotic areas of mouse and human tumors, reaching concentrations 5- to 10-times higher than normal serum levels (Eil et al., 2016). As intact ion transport is essential for T-cell function, high intratumoral K^+^ levels could lead to an impairment of TCR-driven Akt-mTOR phosphorylation. Eil et al. showed that increased K^+^ disturbs Akt-mTOR signaling through the serine/threonine phosphatase PP2A. Importantly, T-cell function could be restored via overexpression of K^+^ channels in T cells, leading to a prolonged survival of tumor-bearing mice (Eil et al., 2016). Thus, the toolbox that tumors might use to evade or modulate anti-tumor immune responses is extensive and future research will determine whether these pathways might provide novel targets for cancer therapy.

## Outlook

The field of cancer immunometabolism gained significant attention in recent years. Taking advantage of the extraordinary amount of data collected in the field of cancer metabolism, experts in immunometabolism were able to apply pre-established techniques and modify pre-existing hypotheses. However, the vast majority of the collected data was obtained in *in vitro* experiments or *in vivo* animal studies. Despite the challenges in experiments involving human samples, such as heterogeneity both within the same tumor and between patients, it is critical to assess the metabolic interaction of immune and cancer cells in human tumors. Nevertheless, several investigators aim to transfer the knowledge from preclinical models to a clinical setting. For example, a recent Phase 1/2 study that assessed the effect of IDO1 inhibition in combination with a DC vaccine showed a good therapy tolerance and suggested a possible chemo-sensitization effect in patients with advanced cancers (Soliman et al., 2018). IDO1 inhibition in combination with checkpoint inhibition (e.g. PD-1 blockade; Box 1) has also been tested in several Phase 1 and 2 studies, with encouraging results. However, recent results from a Phase 3 study that combined the IDO1 inhibitor epacadostat with pembrolizumab, an anti-PD1 antibody, showed that adding epacadostat had no benefit (https://clinicaltrials.gov/ct2/show/NCT02752074).

As the number of mechanisms and thus possible targets is steadily increasing, a key question arises – can modulation of one metabolic pathway, such as through IDO1 blockade, influence the outcome of immune-cancer interaction to induce tumor regression? We speculate that future studies will aim to address the metabolic complexity of the tumor microenvironment rather than target a specific protein or gene. It is currently unclear how the modulation of such a complex system to treat cancer can be achieved. However, exciting diagnostic (e.g. broad gene transcription assessment) or interventional (high-throughput platforms testing compound libraries) technologies are currently emerging and being implemented into laboratory and clinical practice, and might assist in reaching this goal.

This article is part of a special subject collection ‘Cancer Metabolism: models, mechanisms and targets’, which was launched in a dedicated issue guest edited by Almut Schulze and Mariia Yuneva. See related articles in this collection at http://dmm.biologists.org/collection/cancermetabolism.
